# The Alzheimer's Disease-Associated Amyloid β-Protein Is an Antimicrobial Peptide

**DOI:** 10.1371/journal.pone.0009505

**Published:** 2010-03-03

**Authors:** Stephanie J. Soscia, James E. Kirby, Kevin J. Washicosky, Stephanie M. Tucker, Martin Ingelsson, Bradley Hyman, Mark A. Burton, Lee E. Goldstein, Scott Duong, Rudolph E. Tanzi, Robert D. Moir

**Affiliations:** 1 Genetics and Aging Research Unit, Mass General Institute for Neurodegenerative Disease and Department of Neurology, Massachusetts General Hospital, Charlestown, Massachusetts, United States of America; 2 Department of Anatomy and Neurobiology, Boston University School of Medicine, Boston, Massachusetts, United States of America; 3 Department of Pathology, Beth Israel Deaconess Medical Center, Boston, Massachusetts, United States of America; 4 Department of Public Health/Geriatrics, Uppsala University, Uppsala, Sweden; 5 Harvard Medical School, Boston, Massachusetts, United States of America; 6 Molecular Aging and Developmental Laboratory, Photonics Center, College of Engineering, Boston University School of Medicine, Boston University, Boston, Massachusetts, United States of America; 7 Boston University Alzheimer's Disease Center, Boston University, Boston, Massachusetts, United States of America; Mental Health Research Institute of Victoria, Australia

## Abstract

**Background:**

The amyloid β-protein (Aβ) is believed to be the key mediator of Alzheimer's disease (AD) pathology. Aβ is most often characterized as an incidental catabolic byproduct that lacks a normal physiological role. However, Aβ has been shown to be a specific ligand for a number of different receptors and other molecules, transported by complex trafficking pathways, modulated in response to a variety of environmental stressors, and able to induce pro-inflammatory activities.

**Methodology/Principal Findings:**

Here, we provide data supporting an *in vivo* function for Aβ as an antimicrobial peptide (AMP). Experiments used established *in vitro* assays to compare antimicrobial activities of Aβ and LL-37, an archetypical human AMP. Findings reveal that Aβ exerts antimicrobial activity against eight common and clinically relevant microorganisms with a potency equivalent to, and in some cases greater than, LL-37. Furthermore, we show that AD whole brain homogenates have significantly higher antimicrobial activity than aged matched non-AD samples and that AMP action correlates with tissue Aβ levels. Consistent with Aβ-mediated activity, the increased antimicrobial action was ablated by immunodepletion of AD brain homogenates with anti-Aβ antibodies.

**Conclusions/Significance:**

Our findings suggest Aβ is a hitherto unrecognized AMP that may normally function in the innate immune system. This finding stands in stark contrast to current models of Aβ-mediated pathology and has important implications for ongoing and future AD treatment strategies.

## Introduction

The past 25 years has witnessed the accrual of a large body of data concerning the physiochemistry and biological activities of the amyloid β-peptide (Aβ), the main component of β-amyloid deposits in the brains of Alzheimer's disease (AD) patients [1]. Aβ, which is generated in the brain and peripheral tissues, is widely believed an incidental catabolic byproduct of the amyloid β protein precursor (APP) with no normal physiological function. However, Aβ has been shown to be a ligand for a number of different receptors and other molecules [2], [3], [4], transported by complex trafficking pathways between tissues and across the blood brain barrier [1], [5], modulated in response to a variety of environmental stressors, and able to induce pro-inflammatory activities [6], [7]. Despite these clues, the normal physiological role of Aβ remains unknown. We have observed that many of the physiochemical and biological properties previously reported for Aβ are similar to those of a group of biomolecules collectively known as “antimicrobial peptides” (AMPs) which function in the innate immune system. AMPs (also called “host defense peptides”) are potent, broad-spectrum antibiotics that target Gram-negative and Gram-positive bacteria, mycobacteria, enveloped viruses, fungi, protozoans and in some cases, transformed or cancerous host cells. AMPs are also potent immunomodulators that mediate cytokine release and adaptive immune responses (see review by Zaiou, 2007 [8]).

The three main families of mammalian AMPs are the defensins, the histatins, and the cathelicidins. Only one member of the cathelicidin family has been identified in humans, the LL-37 peptide [9]. The pleiotropic LL-37 peptide is a widely expressed archetypal AMP [10]. The rodent LL-37 homologue (CRAMP) has been shown to play a central role in combating bacterial infections in a range of tissues, including the CNS [11]. Patients that express low levels of LL-37 are at increased risk for serious infections [12]. Conversely, high levels of LL-37 are associated with the pathology of several presumably non-infectious diseases [13], including plaques in atherosclerosis [14]. We have observed that LL-37 exhibits striking similarities to Aβ, including a propensity to form cytotoxic soluble oligomers [15], [16], [17], [18] and insoluble fibrils that demonstrate congophilia and birefringence [19], two classical histochemical properties of tinctorial amyloid. While the microbiocidal activity of LL-37 has been well characterized [20], the activity of Aβ against microbial organisms has not been tested.

Here we show that Aβ is active against at least eight common and clinically relevant microorganisms. The *in vitro* antimicrobial activity of Aβ matched, and in some cases, exceeded, that of LL-37, an archetypical human AMP. Furthermore, anti-Aβ immunoreactive material in AD whole brain homogenates is active against *Candida albicans*, the pathogen we identified as most sensitive to synthetic Aβ. Most strikingly, temporal lobe samples from AD brain contained significantly higher antimicrobial activity than material from the same brain area of aged-matched, non-AD subjects. Consistent with an Aβ-mediated action, cerebellum samples with low β-amyloid loads from the same set of affected and unaffected subjects were not significantly different with regards to antimicrobial activity. Our findings show Aβ possesses antimicrobial activity and may function *in vivo* as an AMP and, thus, play a role as an effector molecule of innate immunity.

## Results

Antimicrobial activity against a particular microorganism is measured *in vitro* by a peptide's minimal inhibitory concentration (MIC), which is defined as the lowest concentration able to visibly inhibit growth overnight. We compared the MICs of synthetic LL-37, Aβ40, and Aβ42, against a panel of clinically relevant organisms (**Table 1**). The antimicrobial activity of Aβ peptides was equivalent to or greater than LL-37 for seven of the pathogens tested. These data indicate that Aβ is a *bona fide* AMP with potencies similar to, or, in some cases surpassing those of LL-37. The synthetic Aβ peptides demonstrated antibiotic activity against Gram-negative and Gram-positive bacteria and the yeast *C. albicans*. Activity was isoform-specific for six organisms with Aβ42 showing greater potency compared to Aβ40. Equivalent findings were observed for recombinant Aβ42, material that is free of the potentially toxic contaminants associated with conventional solid-phase peptide synthesis (data not shown). Rodent Aβ42 also demonstrated antimicrobial activity. However, microbial growth was not inhibited by reverse (rAβ42) or scrambled (scAβ42) negative control peptides, thus confirming the antimicrobial action is peptide-specific.

**Table 1:** Aβ peptides possess antimicrobial activity.

			MIC (µg/ml)			
Organism	Aβ42	Aβ40	roAβ42	LL-37	reAβ42	scAβ42
*Candida albicans*	0.78	0.78	0.78	6.25	>25	>50
*Escherichia coli*	1.56	1.56	3.13	1.56	>50	>50
*Staphylococcus epidermidis*	3.13	50	3.13	25	>50	>50
*Streptococcus pneumoniae*	6.25	12.5	6.25	1.56	50	>50
*Staphylococcus aureus*	6.25	25	12.5	6.25	>50	>50
*Listeria monocytogenes*	6.25	25	6.25	25	>50	50
*Enterococcus faecalis*	6.25	50	3.13	6.25	50	>50
*Streptococcus agalactiae*	12.5	50	>50	12.5	>50	>50
*Pseudomonas aeruginosa*	>50	>50	>50	6.25	>50	>50
*Streptococcus pyogenes*	>50	>50	>50	6.25	>50	>50
*Streptococcus mitis*	>50	50	>50	6.25	>50	>50
*Streptococcus salivarius*	>50	>50	>50	50	>50	>50

The antimicrobial activity of synthetic Aβ1-42 (**Aβ42**), Aβ1-40 (**Aβ40**), LL-37 (**LL-37**), reverse **Aβ42-1** (**rAβ42**), or scrambled Aβ42 (**scAβ42**) peptides were determined as minimal inhibitory concentrations (**MIC**) against 12 microorganisms. Antimicrobial activity was assayed by broth microdilution susceptibility test on 96-well plates with microbial growth in wells determined by visual inspection following an overnight incubation. Inhibition of growth in plate wells was confirmed by alamar blue cell viability assay and by surface plating of incubants on agar and counting CFU. Inoculums contained mid-logarithmic phase cells. Consistent with antimicrobial activity specific to the Aβ sequence, inhibition was not observed for reverse and scrambled peptides.

**Figure 1:**
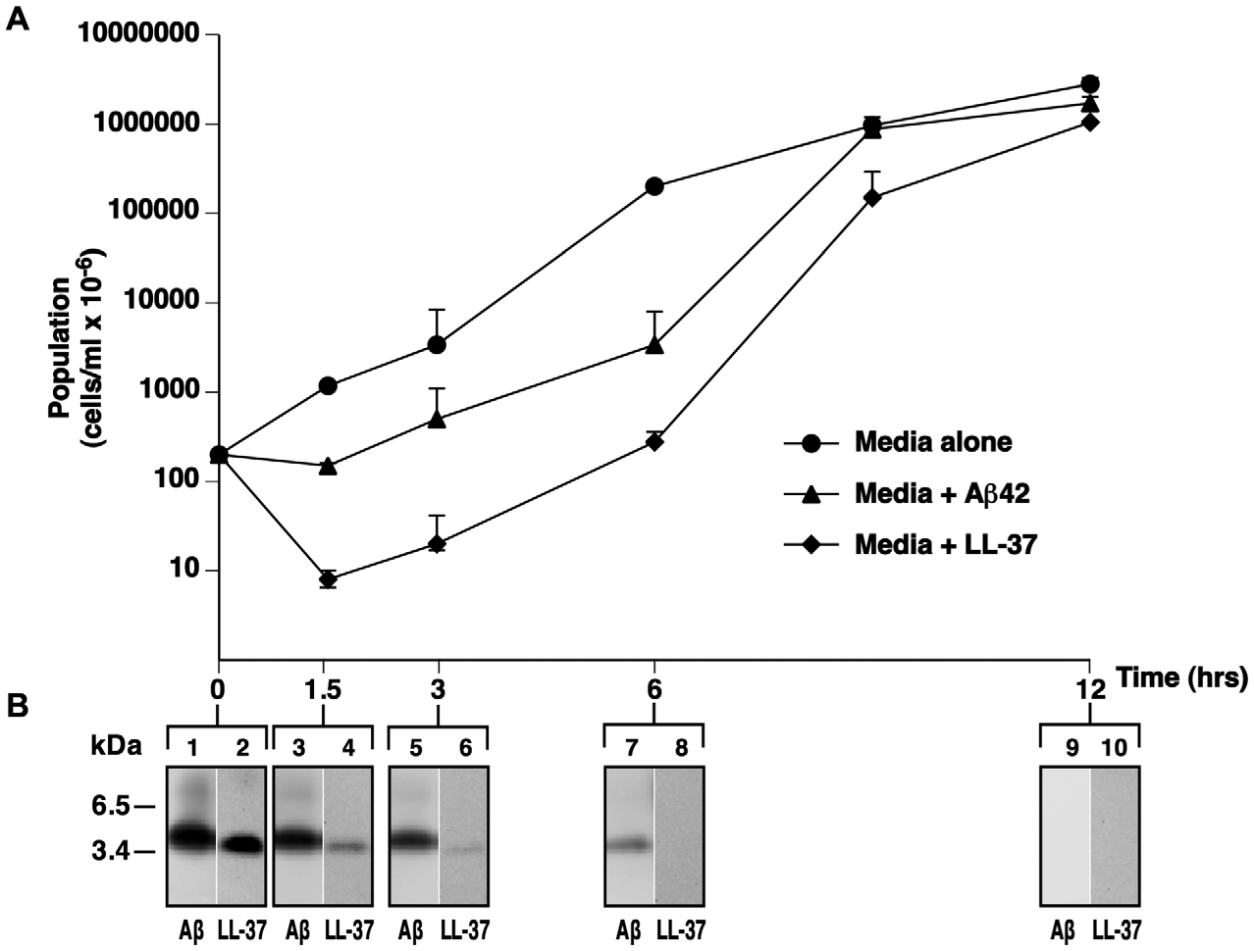
Growth of *E. faecalis* is inhibited by Aβ42. *E. faecalis* were cultured alone (**circle**) with 25 µg/ml of Aβ42 (**triangle**) or LL-37 (**diamond**). **Panel A**; Bacterial growth with time was monitored by inoculation of agar with diluted incubants and counting CFU. Representative data from six experiments is shown as mean signal of four replicates ± s.e.m. **Panel B** Incubants were monitored for Aβ42 and LL-37 by Western blot with mAb 6E10 or anti-LL-37. The figure shows representative signal for Aβ42 (**odd lanes**) or LL-37 (**even lanes**) incubants from six replicate experiments.

AMPs, including LL-37 [21], can be bacteriostatic or bactericidal depending on peptide concentration, ionic strength, and the type of stressor a colony has previously encountered. The growth curves for *E. faecalis* in the presence of Aβ42 suggest a predominantly bacteriostatic action for the peptide against this organism under our incubation conditions (**Figure 1A**). Consistent with previous studies, LL-37 showed potent bactericidal activity against *E. faecalis*. Microbial growth resumed at later time points, most likely due to degradation of LL-37 and Aβ by protective bacterial proteases (**Figure 1B**).

**Figure 2:**
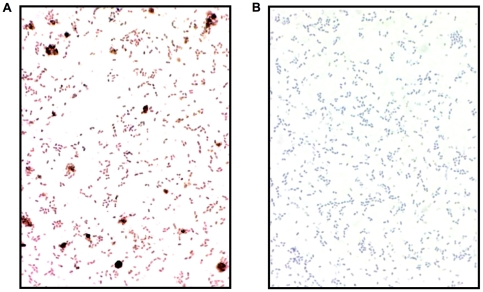
*E. faecalis* pre-incubated with Aβ42 are mAb 6E10 immunoreactive. Bacteria were incubated (1 hr at 37°C) with (**panel A**) or without (**panel B**) Aβ42 (25 µg/ml). Following repeated washes, the bacteria were fixed onto glass slides and immunostained with the HRP conjugated anti-Aβ antibody (mAb 6E10-HRP).

The capacity to associate with microbial lipid bilayers is considered a definitive feature of AMPs [22]. Most antimicrobial peptides are cationic to facilitate binding to anionic bacterial membranes. However, Aβ peptides are anionic under physiological conditions [23]. Nonetheless, data from light microscopic examination of immunostained bacteria pre-incubated with Aβ confirm that the peptide binds to the surface of bacterial cells (**Figure 2**). Binding of Aβ to bacterial membranes is consistent with previous studies showing that Aβ readily binds and disrupts negatively charged synthetic lipid bilayers [24], [25] and anionic mitochondria membranes [26], [27], [28], believed to have been originally derived from bacterial membranes.

**Figure 3:**
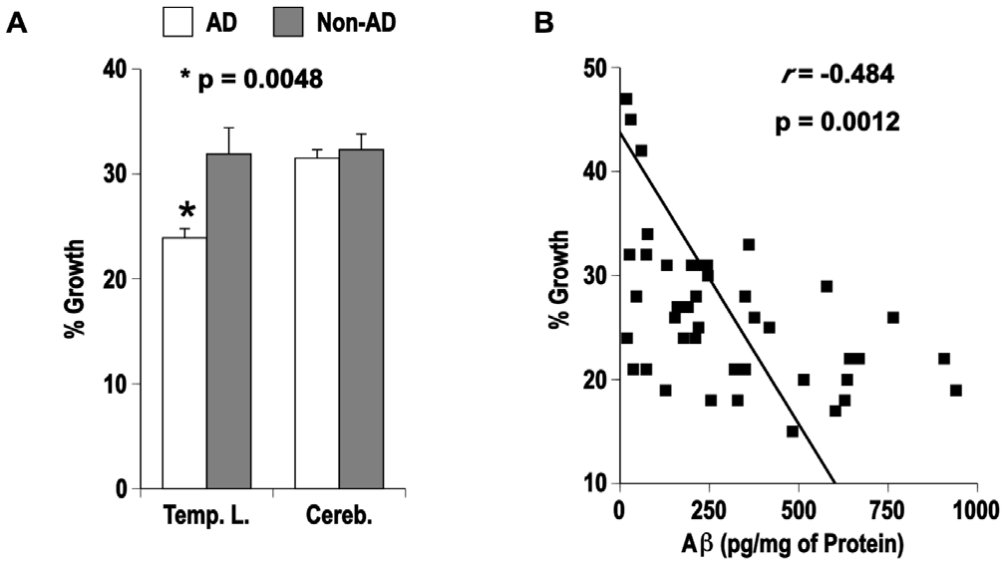
AD brain homogenates have increased antimicrobial activity against *C. albicans*. AD and non-AD brain samples were tested for Aβ-mediated inhibition of *C. albicans*. Samples of temporal lobe (**Temp. L.**) and cerebellum (**Cereb.**) from AD (n = 32) and age-matched control subjects (n = 13) were homogenized in culture broth. **Panel A**; Homogenates were inoculated with log-phase *C. albicans* and microbial growth determined by alamar blue viability assay. Data is shown as percentage of signal for *C. albicans* alone (average of four replicates) ± s.e.m. **Panel B**; Homogenates were assayed for Aβ40 and Aβ42 by commercially available ELISA. Graph shows Aβ signal (sum of Aβ40 and Aβ42) against *C. albicans* growth for temporal lobe homogenates from combined AD and non-demented cohorts (n = 42). Probability analysis used unpaired two-tailed t-tests (**p**). Correlation was determined by calculating the Pearson *r* correlation coefficient (***r***).

**Figure 4:**
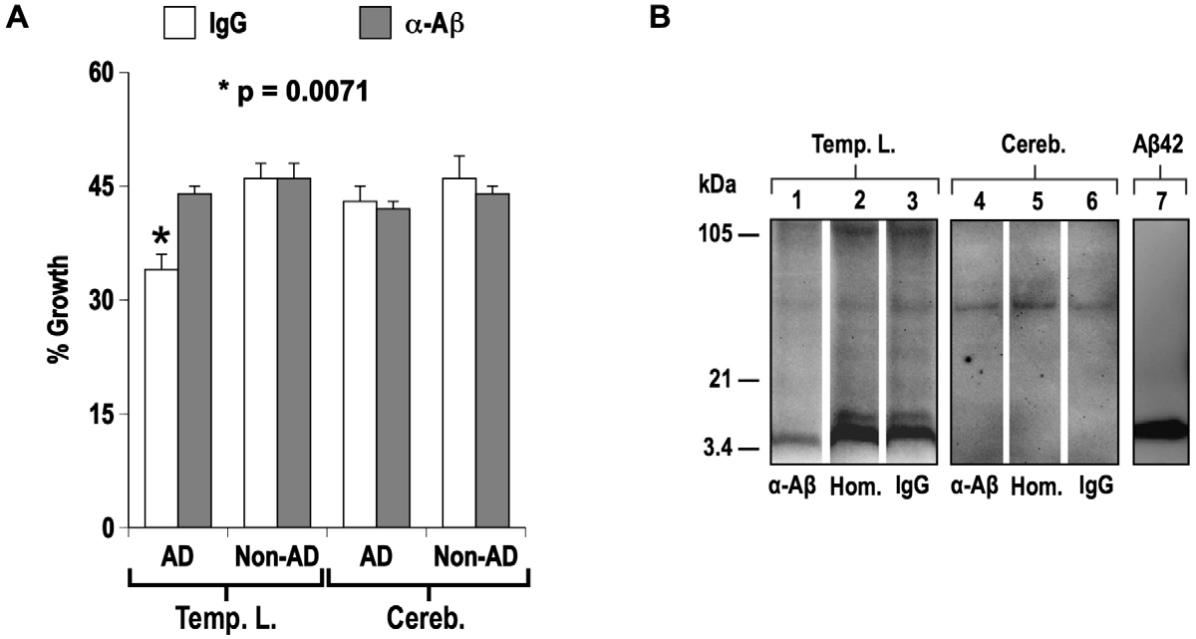
Immunodepletion of Aβ from AD brain homogenates attenuates *C. albicans* inhibition. Homogenates of temporal lobe (**Temp. L.**) and cerebellum (**Cereb.**) were prepared from AD (n = 32) or non-demented (n = 13) subjects. AD (**AD**) or non-demented (**non-AD**) homogenates were pooled and then incubated with Magno-beads pre-loaded with rabbit IgG (**IgG**) or a polyconal rabbit anti-Aβ antibody (**α-Aβ**). Following bead removal samples were analyzed for Aβ signal by Western blot and assayed for *C. albicans* growth by alamar blue viability assay. **Panel A** shows *C. albicans* growth in treated homogenates as a percentage of signal in culture broth alone. Immunodepletion of AD temporal lobe homogenates with α-Aβ restored microbial growth to levels equivalent to non-demented control samples. Graph shows average of five replicates ± s.e.m. **Panel B**; Untreated and immunodepleted homogenates (1∶16 dilution) were Western blotted and probed with the Aβ-specific mAb 4G8 antibody. Analysis confirmed Aβ signal was reduced in temporal lobe homogenate incubated with anti-amyloid β-peptide antibody (**Lane 1**) compared to sample incubate alone (**Lane 2**) or with rabbit IgG (**Lane 3**). Aβ in dilutions of cerebellum homogenate is below the level of detection for our experimental conditions (**Lanes 4–6**). Blots included synthetic Aβ42 (**Aβ42**) standard (**Lane 7**). Statistical probability analysis (**p**) of data used unpaired two-tailed t-test.

In the next experiments we tested if the antimicrobial activity observed for synthetic peptides *in vitro* could be identified in temporal lobe and cerebellum from human brain. Typically β-amyloid load is high in AD temporal lobe and low in cerebellum. Tissue taken from AD (n = 32) or age matched control subjects (n = 13) were homogenized and normalized for protein. Aβ40 and Aβ42 levels in brain homogenates were determined by ELISA. Homogenates were then diluted into culture broth and inoculated with *Candida albicans*. Growth of *C. albicans* was determined using a fluorescence-based alamar blue microplate assay previously described for following cell viability with this organism [29]. AD temporal lobe homogenates inhibited the growth of *C. albicans* significantly more (p = 0.0048) than non-demented control samples (**Figure 3A**). Consistent with an Aβ-mediated antimicrobial activity in AD temporal lobe homogenates, a significant difference in *C. albicans* growth was not observed with cerebellum samples, which carry a considerably lower Aβ load. Also consistent with Aβ-mediated antimicrobial activity, *C. albicans* growth significantly correlated with Aβ concentration in temporal lobe homogenates (**Figure 3B**) but not in cerebellum samples with Pearson's correlation coefficients (*r*) of −0.484, p = 0.0012 and −0.091, p = 0.56, respectively. In addition, the increased antimicrobial activity of AD temporal lobe samples could be significantly attenuated (p = 0.0007) by immunodepletion of homogenates with anti-Aβ antibodies (**Figure 4A**), consistent with an Aβ-mediated antimicrobial activity in AD brain. Analysis of immunodepleted homogenates confirmed Aβ levels were attenuated in samples incubated with rabbit anti-Aβ antibody (**Figure 4B**). Additional experiments confirmed that antimicrobial activity in AD temporal lobe homogenates is also attenuated following immunodepletion with the anti-Aβ mouse monoclonal antibody 6E10 (**Figure S1**).

## Discussion

Aβ peptides inhibited the growth of eight of 12 clinically important pathogens screened (**Table 1**), including the bacteria *S. pneumoniae*, which is a leading cause of bacterial meningitis [30], and *C. albicans*, the most common cause of neurocandidiasis [31]. If the normal function of Aβ is to function as an AMP, then an absence of the peptide may result in increased vulnerability to infection. Such an association has been shown for LL-37 and the disorder morbus Kostmann in which patients deficient in this AMP cannot mount an effective defense against pathogens [32]. To our knowledge a relationship between human immunodeficiency and low Aβ levels has not been investigated. However, knockout mice that lack the proteases that generate endogenous rodent Aβ appear to have increased susceptibility to pathogens [33]. BACE1 knockout (KO) mice that generate low levels of Aβ and BACE1- and BACE2-deficient double KO mice, which do not express Aβ, have mortality rates of 40 and 60 percent, respectively. Housing the animals in a pathogen-free environment restores survival rates to that of wild-type mice (>95 percent). The etiology of the immunodeficiency has been investigated but not identified. Adaptive immune responses to vesicular stomatitis virus are the same for BACE-KO and wild-type mice. In addition, markers for adaptive immune system function are normal in BACE-KO mice, including leukocytes migration into the peritoeum following thioglycolateacute-induced acute peritonitis and T-cell cytotoxiocity towards non-host cells. More recently, in a clinical trial of the Aβ42- lowering agent tarenflurbil patients receiving the drug have significantly increased rates of infection [34]. Increased pathogen susceptibility of apparently adaptive immunocompetent BACE-KO mice and AD patients with suppressed Aβ expression is consistent with our finding that Aβ may have a normal protective function as an antimicrobial peptide of the innate immune system.

The immunostatus of APP knockout (APP-KO) mice has yet to be characterized. APP is a member of a larger protein family that includes the amyloid protein precursor-like proteins 1 and 2 (APLP1 and APLP2) [35], [36]. APP and APLP proteins appear to have overlapping and partially redundant functions [37], [38], [39] and share processing pathways, including BACE-mediated generation of APLP-derived peptides analogous to Aβ [37], [38], [39], [40]. It is unclear to what degree APLP-derived proteins may compensate for deficiencies associated with low APP expression. Mice lacking both APP and APLP proteins (triple APP/APLP KO-mice) show early postnatal mortality with severe developmental abnormalities [41], [42], [43], [44], [45]. Interestingly, local cortical dysplasias can be infection-mediated and are observed in 68% of triple APP/APLP KO-mice [45]. Partial penetrance is also suggestive of an environmental component in this ectopia.

Recent studies have shown that while the adaptive immune system has limited access to the brain, the CNS can still mount a robust response to invading pathogens via antimicrobial peptides and the innate immune system. Numerous innate immune molecules with potent antimicrobial activity are found in brain, including the recently identified chromogranins [46], neuropeptides neurokinin-1, enkelytin and peptide B, neuropeptide Y, polypeptide tyrosine-tyrosine, and the peptide hormones α-melanocyte stimulating hormone, adenoregulin, adrenomedullin and proadrenomudullin, corticostatin RK-1, neurotensin, and bradykinin [47]. Consistent with an antimicrobial role for brain generated Aβ, we found AD temporal lobe homogenates contain an average of 24% greater activity against *C. albicans* than samples from non-AD subjects (**Figure 3A**). Furthermore, higher Aβ levels in temporal lobe samples correlated with increased inhibition of *C. albicans* (**Figure 3B**) while immunodepletion of Aβ from AD brain homogenates restored antimicrobial activity to levels equivalent to those of control homogenates (**Figures 4A** **and S1**). Immunoblot analysis confirmed attenuated Aβ levels in anti-Aβ antibody immunodepleted samples and low β-amyloid load in cerebellum tissue (**Figure 4B**). These data support a protective role for Aβ under the conditions found in the brain milieu even though *in vivo* concentrations of soluble peptide are substantially lower than levels in experiments using synthetic peptide [48]. Several factors may contribute to this apparent discrepancy. First, synergistic AMP interactions *in vivo* potentiate antimicrobial activity [49]. This effect has been demonstrated for CRAMP (rodent LL-37), for which peptide levels in rodent CNS do not approach concentrations that are needed to obtain positive signals in *in vitro* assays. However, rat brain extracts depleted of CRAMP have substantially attenuated antimicrobial activity [50]. Moreover, mutant mice lacking CRAMP are more susceptible to CNS infection by *meningococcal meningitis* [11]. Second, AD brain contains a large pool of neurotoxic oligomeric Aβ species [51], [52]. Oligomerization plays a key role in the targeting and permeabilization of bacterial membranes by AMPs [19], [53], [54]. Neurotoxic oligomeric Aβ species present in AD brain may enhance the antimicrobial activity of homogenates beyond that predicted from *in vitro* experiments, which add synthetic monomeric peptides to microbial cultures.

A large body of data supports a central role for neuroinflammation in AD neuropathology [55]. A number of studies have proposed Aβ as the source of AD-associated inflammation [56]. However, a re-evaluation of the role of Aβ in inflammation may now be warranted in view of these data suggesting that the peptide functions as an AMP in tissues. Inflammatory response in the immunologically privileged CNS is mediated by the innate immune system. Rather than Aβ acting as a sole independent initiator of neuroinflammation, our data raise the possibility that the peptide may be part of a response mounted by the innate immune system. Thus, Aβ may be one of a family of AMPs known to contribute pro-inflammatory activities under disease conditions. At least one other disease has been shown to involve deposition of an AMP as amyloid, corneal amyloidosis. In corneal amyloidosis the widespread and well-characterized antimicrobial protein lactoferrin accumulates in the subepithelium as insoluble amyloid [57], [58]. Semenogelin-derived antimicrobial peptides [59] are also deposited as seminal vesicle amyloid [60] in a common sub-clinical pathology found in elderly men [61]. Based on our current findings, we postulate that stimulation of the innate immune system may initially trigger Aβ generation and the β-amyloid cascade that leads to β-amyloid deposition. Along these lines at least three pathogenic mechanisms could conceivably lead to Aβ generation and accumulation in the CNS via stimulation of an innate immune response. First, persistent sub-acute CNS infection may drive chronic activation of the innate immune system. A number of studies have reported that the CNS of AD patients is infected with pathogens including *Chlamydia pneumoniae* [62], *Borrelia spirochetes* [63], *Helicobacter pylori* [64], and HSV [65]. Deposition of β-amyloid has also been reported for acquired immunodeficiency syndrome patients with brain HIV infection [66]. Given the known genetic influence on Aβ accumulation, genetic factors may contribute to activation of the innate immune system by regulating Aβ production and clearance. At one of the end of the spectrum of known AD genes, highly penetrant mutations such as those in the early-onset familial AD genes, *APP*, *PSEN1*, and *PSEN2*, would constitutively trigger cerebral Aβ accumulation with no need for activation of the innate immune system [67]. At the other end of the spectrum, consistent with the increase risk of AD associated with the *ε4* variant of the apolipoprotein E gene [68], carriers of the *ε4* allele are reported to have higher rates of CNS infection for several of these pathogens [69]. Finally, in a recent family-based genome-wide association scan for late-onset AD, one of four genes achieving genome-wide significance for association with AD was a homologue of CD33, a lectin involved in the innate immune system [70].

In a second potential pathogenic mechanism, a transient infection may lead to a self-perpetuating innate immune response. Transient triggers may include pathogens reported to be present in AD brain. And in a third mechanism, an inappropriate inflammatory response by the innate immune system to transient or persistent non-infectious insults could also trigger a self-perpetuating innate immune response. While dozens of diseases have been suggested to involve immune abnormalities, for most, the underlying cause of the aberrant immunoresponse remains unclear. For AD, traumatic brain injury [71], stroke [72] and certain forms of inhalant anesthetics [73] have been linked to increased cerebral Aβ levels. Thus, while an infection-mediated pathological mechanism for AD is certainly one possibility for triggering an innate immune response in the CNS and subsequent production of antimicrobially active Aβ, other non-microbial factors may also be involved. Interestingly, peptides containing the microtubule binding sites on tau proteins have also been shown to harbor antimicrobial properties [74].

The capacity to associate with lipid bilayers is considered a definitive feature of AMPs, and the peptides usually affect their antimicrobial activity by membrane permeabilization [75]. Membrane disruption is also thought to be a mechanism for Aβ-mediated cytotoxicity [24], [26]. Our finding that bacterial membranes stain positive for Aβ following incubation with the peptide (**Figure 2**) is consistent with a mechanism that involves association with microbial lipid bilayers. While most AMPs are cationic, Aβ peptides are anionic. Repulsive electrostatic forces between anionic peptides and electronegative phospholipids in bacterial membranes potentially limit antimicrobial activity of this class of AMP. However, in addition to our data, previous studies have conclusively shown that Aβ readily binds and disrupts both synthetic anionic lipid bilayers [24] as well as mitochondrial membranes [26]. Interestingly, mitochondria are thought to be of endosymbiont origin and have anionic membranes that resemble the lipid bilayers of bacteria. A number of AMPs, including LL-37, appear to target and disrupt the mitochondrial membranes of parasitic protozoans [8]. Recent studies have also identified a number of anionic mammalian peptides with antimicrobial activity, including CNS neuropeptides [76] and peptide hormones [47]. Structural studies on the important epithelial anionic AMP dermicidin have shown that an overall positive charge is not a prerequisite for binding of bacterial membranes [77]. Rather, the key modulators of lipid bilayers/peptide association are the peptides charge distribution and secondary conformation. Collectively, these data indicate that AMP activity is not limited to cationic species and that anionic peptides such as Aβ can readily bind bacterial membranes and act as potent antimicrobial agents.

In *E. faecalis* cultures, Aβ was more resistant to bacterial-mediated degradation than LL-37 (**Figure 1B**). Bacterial defense mechanisms secrete proteases that target positively charged peptides. Anionic AMPs are believed to be, at least in part, a host counter measure to bacterial resistance mechanisms [78]. Oligomerization is also thought to protect AMPs from microbially-mediated degradation, and Aβ oligomers have been shown to be highly protease resistant. An anionic charge and propensity to oligomerize may therefore help render Aβ resistant to bacterial attack.

AMPs cytotoxicity is usually highly specific for microbes. However, AMPs can also be cytotoxic to select host cells under physiological conditions. Host cell cytotoxicity has been shown for LL-37 [14] which, like Aβ [79], is cytotoxic towards vascular smooth muscle cells. AMP host cell cytoxicity often involves disruption of mitochondrial function, an activity reported for both LL-37 [80], [81] and Aβ [27]. Thus, neurotoxicity that has been shown for Aβ is consistent with AMP behavior. The role of AMP host cell cytoxicity in disease and defense is unclear. LL-37 cytotoxicity has been implicated in disease pathology [14] but may also have a normal function in antibody-dependent cell cytotoxicity, a host mechanism for the clearance of virus-infected and transformed cells [81]. At present Aβ's host cell cytotoxicity is only associated with disease. Identification of Aβ as an AMP raises the possibility that host cell cytotoxicity, or at least a component of this activity, may also have a role in innate immunity.

In summary, our finding that Aβ is an antimicrobial peptide is the first evidence that the species responsible for amyloidosis may have a normal function. This stands in stark contrast to current models, which assume β-amyloid deposition to be an accidental process resulting from the abnormal behavior of an incidental product of catabolism. Our data suggest increased Aβ generation, and resulting AD pathology, may be a mediated by a response of the innate immune system to a perceived infection. This model has important implications for current and future AD treatment strategies. First, it raises the possibility of preventing amyloidosis from initiating by pre-emptive targeting of pathogens/insults that stimulate the brain's innate immune system. Second, our model identifies the inflammatory pathways of the innate immune system as targets for modulating Aβ generation/accumulation. The target pathways implicated here are downstream of the inflammatory trigger. Thus, this approach would likely be useful independently of the involvement of infectious agents in AD pathology.

## Materials and Methods

### Synthetic Peptides

Experiments used Aβ1-40 (Aβ40), Aβ1-42 (Aβ42), scrambled Aβ (scAβ42), Aβ42-1 (rAβ42), LL-37, and scrambled LL-37 (scLL-37) peptides. Aβ and LL-37 peptides were prepared and purified by Dr. James I. Elliott at Yale University (New Haven, CT) using solid-phase peptide synthesis. Scrambled LL-37 peptide was from AnaSpec (San Jose, CA). Recombinant human Aβ42 (recAβ42) and rodent Aβ42 (roAβ42) were purchased from rPeptide (Bogart, GA) and Calbiochem (Gibbstown, NJ) respectively. Findings for recombinant and SPPS prepared peptides were equivalent in all experiments.

### Brain Samples

Human brains were obtained 12–24 hrs postmortem. At the time of autopsy, one cerebral hemisphere was sectioned and frozen at −70°C and the other hemisphere was fixed in formalin for histological examination. The clinical diagnosis of AD was confirmed by subsequent histological evidence of amyloid plaques and neurofibrillary tangles. Samples were provided by the Neurobiology Tissue Bank at the Mass General Institute for Neurodegenerative Disease and Massachusetts General Hospital and included temporal lobe and cerebellum from 32 AD patients and 13 non-demented age-matched control subjects.

### Cell Cultures

Bacteria were from the American Type Culture Collection (ATCC, Manassas, VA) and included *Candida albicans* ATCC 10231, *Escherichia coli* ATCC 25922, *Staphylococcus epidermidis* ATCC 12228, *Streptococcus pneumoniae* ATCC 49619, *Staphylococcus aureus* ATCC 25923, *Listeria monocytogenes* ATCC 19112, *Enterococcus faecalis* ATCC 29212, *Streptococcus agalactiae* ATCC 12386, *Pseudomonas aeruginosa* ATCC 27853, *Streptococcus pyogenes* ATCC 19615, *Streptococcus mitis* ATCC 6249, and *Streptococcus salivarius* ATCC 13419. Bacteria were cultured aerobically in Mueller-Hinton broth (MHB), Brain and Heart Infusion broth (BHIB), or BHIB supplemented with 1% lysed horse blood and plated on Tryptone Soy Agar (TSA) plates containing 5% defibrinated sheep blood. *C. albicans* was grown in RPMI-1640 medium (Hyclone, Logan, UT) with 2% glucose buffered (pH 7.0) and 0.165 M MOPS and surface plated on sabouraud dextrose agar plates. Culture conditions for each organism are included in **Table S1**. Organisms were subcultured for 2 hrs to generate mid-logarithmic growth cultures for use as inoculates in experiments. Media reagents were obtained from Becton, Dickinson and Company (Sparks, MD).

### Preparation of Inoculum Containing Mid-Logarithmic Phase Cells

Colonies from agar were transferred by sterile loop to growth media and incubated for 2 hrs at 37°C to achieve a McFarland density of 0.5. Bacteria inoculum cell densities were normalized to 5×10^5^ cells/ml immediately before use photometrically and subsequently confirmed by colony count. Inoculum of *C. albicans* contained a cell density of 2.5×10^3^ CFU/ml.

### Peptide Pre-Treatment and Preparation of Stock Solutions

Bulk powdered peptides were first dissolved in 30% trifluoroethanol (TFE) at 1 mg/ml. Five hundred microliter aliquots of the stock solutions were lyophilized and stored under nitrogen at −20°C. Stock solutions at 2 mg/ml were prepared the day of experimentation from the peptide films by solubilizing a second time in either water or 20% TFE. Aβ stocks prepared in water were sonicated and insoluble peptide aggregates pelleted by centrifugation (10 min×16,000 *g*). Peptide concentrations in stock solutions were determined immediately before use by bicinchoninic acid (BCA) protein assay. The validity of BCA for assaying Aβ peptides has been established previously [82]. For MIC experiments, peptides were serially diluted into growth media. For other experiments stocks were diluted into required working buffers. Experiments included controls for peptide buffer vehicle alone.

### MIC Determination

Peptide antimicrobial activity was determined as minimal inhibitory concentration (MIC) [83]. Experiments identified peptide MIC by broth microdilution susceptibility test in conjunction with CFU and alamar blue assays. Inoculum containing mid-logarithmic phase cells was dispensed into the wells of polypropylene 96-well plates (Fisher, Pittsburgh, PA) containing seven two-fold dilutions of test peptide in growth media. Plates were then incubated aerobically overnight (12 to 18 hrs) at 37°C. Peptide MIC was taken as the lowest concentration able reduce cell growth by CFU and alamar blue assays by at least two-fold and which correlated with the visible loss of a growth button on the bottom of microtiter wells. Experiments were repeated a minimum of three times for each organism, and tests included at least three replicates for each assay condition. Experiments included control serial dilutions of buffer vehicle alone.

#### Note on radial diffusion assays (RDAs)

RDAs have been widely used in previous studies to assess AMP antimicrobial activity. However, in our experiments RDAs proved unreliable for testing Aβ antimicrobial activity because the peptide failed to diffuse away from the point of application (**data not shown**). Aβ solutions are prone to aggregation, particularly in the presence of even trace amounts of metal, and interaction with contaminates or the media matrix may lead to rapid precipitation of the peptide within the agar. Aβ peptides also appear to irreversibly absorb to the cellulose disks often used as sample reservoirs in RDAs.

### CFU Assay

Serial dilutions of incubants were prepared and streaked onto the surface of agar. The agar plates were then incubated overnight at 37°C and colonies forming units counted.

### Alamar Blue Cell Viability Assay

Microbial growth was determined by following the reduction of the synthetic metabolic substrate resazurin (alamar blue) to a fluorescent product by respiratory enzymes in living cells [84]. Alamar blue assay is used in high throughput screens for antimicrobial agents [85] and is available commercially in kit form from Invitrogen. Microbial growth in experiments was assayed with alamar blue kits according to the manufacturer's instructions. Briefly, resazurin reagent was added to microbial cultures (1∶10) and samples incubated for 30 or 60 minutes. Fluorescence signal was measured at excitation of 530 nm and emission at 590 nm. Signal was blanked on sterile media. For experiments with brain homogenates, blank wells contained all components as tests but were not inoculated with *C. albicans*.

### Anti-Aβ Imunostaining of Bacteria

*E. faecalis* smears were air-dried on Superfrost/Plus microscope slides (Fisher Scientific, Pittsburgh, PA) and then heated to kill and fix bacterial cells. Fixed cells were incubated with 3% methanolic hydrogen peroxide for 30 minutes at room temperature to inhibit endogenous peroxidase activity, passed through graded alcohol, and rinsed three times in deionized water and phosphate-buffered saline (PBS). Slides were then incubated with a 1∶2,000 dilution of the anti-Aβ monoclonal antibody (mAb) 6E10 (Covance, Princeton, NJ) in TBST. Following washing, slides were incubated with goat anti-mouse IgG-coupled to HRP (1∶200). Detection and localization steps were performed using Vectastain ABC kit and DAB Substrate Kit (Vector Laboratories, Burlingame, CA).

### Assaying Antimicrobial Activity in Brain Homogenate

Samples of AD (n = 32) or non-demented control (n = 13) temporal lobe and cerebellum were homogenized in three volumes of 10 mM phosphate buffer, pH 7.4 by 12 passes in a glass-on-Teflon homogenizer. Homogenates were diluted into RPMI-1640 media to inhibit *C. albicans* growth by approximately 50 percent (**Figure S1**). Samples were then inoculated (2.5×10^3^ CFU/ml) with mid-logarithmic growth culture of *C. albicans* and incubated aerobically for 3 hrs at 37°C in 96-well microplates (100 µl/well). Alamar blue reagent was added to wells (10 µl) and fluorescence measured after 30 and 60 minutes incubation. Signal from test wells was blanked on samples incubanted without *C. albicans*. Signal from homogenate blanks was equivalent to uninoculated media alone (**data not shown**). Samples were assayed in quadruplicate.

### Assaying Aβ in Tissue Homogenates

Aβ40 and Aβ42 in samples were determined using commercially available ELISA kits (Covance, Princeton, NJ). Brain homogenates were assayed according to the manufacturer's instructions.

### Immunodepletion of Brain Homogenates

MagnaBind goat anti-rabbit IgG beads (Pierce, IL) were pre-incubated overnight with the Aβ specific rabbit anti-amyloid β-peptide antibody (Invitrogen, CA) or rabbit IgG then washed repeatedly. Pooled samples were prepared from temporal lobe (30 AD and 12 non-AD) or cerebellum (32 AD and 13 non-AD) homogenates. The pooled brain homogenates were incubated alone or with the antibody loaded beads at 4°C for 2 hrs. Final incubation conditions were 5 µg of antibody per mg of original tissue (w/w). Beads were pelleted and soluble fraction removed. Fractions were immunoblotted and probed with the Aβ specific mAb 4G8 (Covance, Princeton, NJ). Analysis confirmed anti-Aβ antibody treated homogenates were depleted of Aβ (**Figure 4B**). Soluble fractions were then tested for antimicrobial activity against *C. albicans* by alamar blue assay.

### Immunoblotting (Western Blotting)

Samples were first resolved by electrophoresis on SDS-PAGE (4–12% Bis-Tris gels) and then transferred to polyvinylidene fluoride membrane. Membranes were blocked with bovine serum albumin (10%) then probed with mAb 4G8 (1∶200), mAb 6E10 (1∶2,000), or mAb anti-LL-37 (1∶200) (Hycult Biotechnology, Uden, The Netherlands). Following washing, membranes were incubated with goat anti-mouse IgG-coupled to HRP. Blots were developed with chemiluminescence reagent (Pierce, Rockford IL) and signal captured using a VersDoc digital imaging system (BioRad, Hercules, CA). Blot incubations used Tris buffered saline, pH 8 containing 0.1% Tween (TBST).

### Statistical Analysis

Association coefficients between Aβ levels in brain homogenate and *C. albicans* growth were calculated using Pearson correlation test and linear regression. Experimental groups were compared by unpaired two-tailed t-test with a nominal alpha criterion level of 0.05. Antimicrobial signal in AD and non-AD cohorts passed a D'Agostino-Pearson test for normality (alpha = 0.05) with p values of 0.077 and 0.24, respectively. Variances of signal from AD and non-AD cohorts were not significantly different (p = 0.18). Alternative non-parametric statistical analysis of antimicrobial activity in temporal lobe homogenates by two-tailed Mann-Whitney U test also returned a significant difference between AD and non-AD cohorts (p = 0.018). Statistical analysis used GraphPad Prism software package (La Jolla, CA).

## Supporting Information

Figure S1 Aβ-mediated inhibition of *C. albicans* in AD brain homogenates is dose dependant. AD temporal lobe (Temp. L.) or cerebellum (Cereb.) were homogenized in phosphate buffer. Temporal lobe (n = 30.) or cerebellum (n = 32) homogenates were pooled and 1∶16, 1∶32, and 1∶64 serial dilutions prepared in culture broth. Homogenate dilutions were incubated with mouse IgG (IgG) or anti-Aβ mAb 6E10 (6E10) antibody immobilized on MagnaBind beads. Following pelleting of the beads incubants were inoculated with mid-logarithmic phase *C. albicans* in 96-well plates. Microbial growth was determined by alamar blue cell viability assay. Graphs shows percentage signal of *C. albicans* alone (average of five replicates) ± s.e.m. Consistent with Aβ-mediated antimicrobial activity, *C. albicans* growth is highest for samples with low Aβ levels and increases with homogenate dilution.
(0.10 MB TIF)

Table S1 Experimental culture conditions for test organisms. The table shows test organisms used for peptide MIC determination with Gram staining (Gram Stain) properties, American Type Culture Collection designation (ATCC No.), culture media (Growth Media), and growth period (Incub. hrs) used for broth microdilution susceptibility testing. Organisms were grown aerobically at 37°C in Mueller-Hinton broth (MHB), Brain and Heart Infusion broth alone (BHIB) or supplemented with 1% lysed horse blood (BHIB/LHB), or RPMI-1640 medium with 2% glucose (RPMI-1640).
(0.05 MB PDF)
