# Supplementary material for: The Alzheimer's Disease-Associated Amyloid β-Protein Is an Antimicrobial Peptide
Source: PLoS One. 2010 Mar 3;5(3):e9505. doi: 10.1371/journal.pone.0009505 (PMC2831066; doi:10.1371/journal.pone.0009505)
Supplement: Table S1 — Experimental culture conditions for test organisms. The table shows test organisms used for peptide MIC determination with Gram staining (Gram Stain) properties, American Type Culture Collection designation (ATCC No.), culture media (Growth Media), and growth period (Incub. hrs) used for broth microdilution susceptibility testing. Organisms were grown aerobically at 37°C in Mueller-Hinton broth (MHB), Brain and Heart Infusion broth alone (BHIB) or supplemented with 1% lysed horse blood (BHIB/LHB), or RPMI-1640 medium with 2% glucose (RPMI-1640). (0.05 MB PDF) [file pone.0009505.s002.pdf]

Organism	Gram Stain	ATCC No.	Growth Media	Incub. (hrs)
Staphylococcus aureus	+	25923	MHB	12
Escherichia coli	-	25922	BHIB	12
Listeria monocytogenes	+	19112	BHIB	18
Pseudomonas aeruginosa	-	27853	BHIB	18
Enterococcus faecalis	+	29212	BHIB	18
Staphylococcus epidermis	+	12228	BHIB	18
Streptococcus agalactiae	+	12386	BHIB/LHB	12
Streptococcus pneumoniae	+	49619	BHIB/LHB	12
Streptococcus mitis	+	6249	BHIB/LHB	12
Streptococcus pyogenes	+	19615	BHIB/LHB	18
Streptococcus salivarius	+	13419	BHIB/LHB	12
Candida albicans		10231	RPMI-1640	18
